# Looking back: twenty years of reforming undergraduate medical training and curriculum frameworks in Switzerland

**DOI:** 10.3205/zma001272

**Published:** 2019-10-15

**Authors:** Raphael Bonvin, Mathieu Nendaz, Peter Frey, Kai P. Schnabel, Sören Huwendiek, Christian Schirlo

**Affiliations:** 1 Universität Fribourg, Unité Pédagogie Médicale, Fribourg, Switzerland; 2 Hôpitaux Universitaires Genève, Institut de médecine de premier recours, Genève, Switzerland; 3 Universität Bern, Medizinische Fakultät, Studiendekanat, Bern, Switzerland; 4 Universität Bern, Institut für medizinische Lehre, Abteilung für Unterricht und Medien, Bern, Switzerland; 5 Universität Bern, Institut für medizinische Lehre, Abteilung für Assessment und Evaluation AAE, Bern, Switzerland; 6 Universität Zürich, Geschäftsstelle Direktorium UMZH, Medizinische Fakultät, Geschäftsbereich Struktur & Entwicklung, Zürich, Switzerland

**Keywords:** curriculum design, innovation, study reform, national licensure exam, curricular changes, undergraduate training, framework of reference, outcome-based education

## Abstract

**Introduction:** To date, hardly any reports exist that outline the reforms in medical studies in Switzerland from the first partial reforms in the 1970s until today.

**Methods: **This article outlines the recent history of medical curricula, their reforms in the early 1970s and, based on these, the key reasons for the major curricular reforms of the 2000s from the perspective of the authors.

**Results: **The various projects, initiatives and legislative elements at the national level include the introduction of new quality control instruments – federal examination and programme accreditation, the introduction of a national catalogue of learning objectives and its two follow-up editions, as well as the implementation of the Bologna reform in undergraduate medical curricula. Examples of the key new elements found in all medical training in Switzerland include: the interdisciplinary orientation of learning content in organ and functional system-oriented subject areas or modules, the enhanced valorisation of practical clinical training, as well as the introduction of problem-oriented formats and the integration of partly formative, partly summative exams according to the format of the objective structured practical examination (OSCE). Characteristics unique to the four medical faculties and their medical training programme are also highlighted.

**Discussion: **The described projects, initiatives and legislative elements have led to a dynamic, continuous development of medical curricula in Switzerland. The close cooperation between the faculties and the Federal Office of Public Health (FOPH) has also resulted in a redefinition of the roles and responsibilities of universities and the Federal Government according to the new Law on Medical Professions. This guarantees the medical faculties a great deal of autonomy, without neglecting quality assurance.

## Zusammenfassung

**Einleitung:** Bisher existieren kaum Berichte, die die Schweizer Reformen des Medizinstudiums von den ersten Teilreformen in den 1970er Jahren bis heute skizzieren.

**Methoden: **In der vorliegenden Arbeit werden kursorisch die jüngere Geschichte der humanmedizinischen Curricula, deren erste Teilreformen in den frühen 1970er Jahren und darauf aufbauend die wesentlichen Ausgangspunkte für die grossen Curriculumsreformen der 2000er Jahre aus Sicht der Autoren beschrieben.

**Ergebnisse: **Die vielfältigen Projekte, Initiativen und gesetzgebenden Elemente auf Eidgenössischer Ebene umfassen die Einführung von neuen Qualitätsentwicklungsinstrumenten – Eidgenössische Prüfung und Programmakkreditierung, die Einführung und Weiterentwicklung eines nationalen Lernzielkataloges über insgesamt drei Editionen sowie die Einführung der Bologna Reformen auch in den humanmedizinischen Studiengängen.

Im Sinne der Entwicklung von ausdifferenzierten Modellstudiengängen können exemplarisch die wesentlichen neuen Elemente aller Studiengänge in der Schweiz charakterisiert werden: die interdisziplinäre Ausrichtung der Lerninhalte in organ- und funktionssystem-orientierten Themenblöcken oder Modulen, die Aufwertung der klinisch-praktischen Ausbildung sowie die Einführung von problem-orientierten Formaten und der Integration von teils formativen, teils summativen Prüfungen nach dem OSCE-Format. Aufgezeigt werden auch die besonderen standort-spezifischen Charakteristika von vier Medizinischen Fakultäten und deren humanmedizinischen Studiengängen.

**Diskussion: **Die beschriebenen Projekte, Initiativen und gesetzgebenden Elemente haben in der Schweiz zu einer dynamischen, weiterhin anhaltenden Entwicklung der humanmedizinischen Curricula geführt. Die enge Zusammenarbeit zwischen den Fakultäten und dem Bundesamt für Gesundheit (BAG) hat zudem bewirkt, dass mit dem neuen Medizinalberufegesetz Rollen und Verantwortungen zwischen Universitäten und Bund neu definiert worden sind. Dies gewährleistet den Fakultäten eine grosse Autonomie ohne die Qualitätssicherung zu vernachlässigen.

## 1. Introduction

In the last 20 years, the context of medical education in Switzerland has undergone profound changes. The Federal Government departed from a system of heavily centralised control based on a uniform intra-university examination programme and handed over full autonomy of teaching and examinations to the medical faculties. The supervisory role of the Federal Government is executed via three instruments: programme accreditation, a catalogue of learning objectives and a licensing exam. This article aims to provide a better understanding of the background and current developments in this training landscape and to discuss successful and inhibitory factors in curricular reforms. 

## 2. Methods

All six Swiss medical faculties were invited to contribute to this retrospective account of the development of undergraduate medical education over the past 20 years. This article presents the joint work of four faculty representatives who responded to this call. All authors have been involved in these changes at various levels, some since the end of 1990 and the others since the beginning of the 2010s. They present a short historical account of the latest developments in Swiss medical university education from their perspective, as well as the changes in their local curricula.

## 3. Results

This retrospective account is divided into three parts. First, the context in which the current changes took place is outlined; then the relevant changes at the federal level are described, and finally, four undergraduate curricula illustrate how the changes were implemented at the faculty level.

### 3.1. The background to the reforms of the 2000s

Medical education in Switzerland has been governed by federal law since 1877. Until 2007, this law stipulated which exams should be taken in each academic year, thus dictating a largely uniform curriculum throughout Switzerland. The last profound revision of this federal law was implemented in 1972 (the so-called “Rossi Plan”); for the first time, this also affected teaching methods (and not only exams): The pre-clinical phase was shortened from five to four semesters, clinical rotations were introduced in the fourth academic year and federal exams began using multiple-choice questions. 

**Figure 1:**
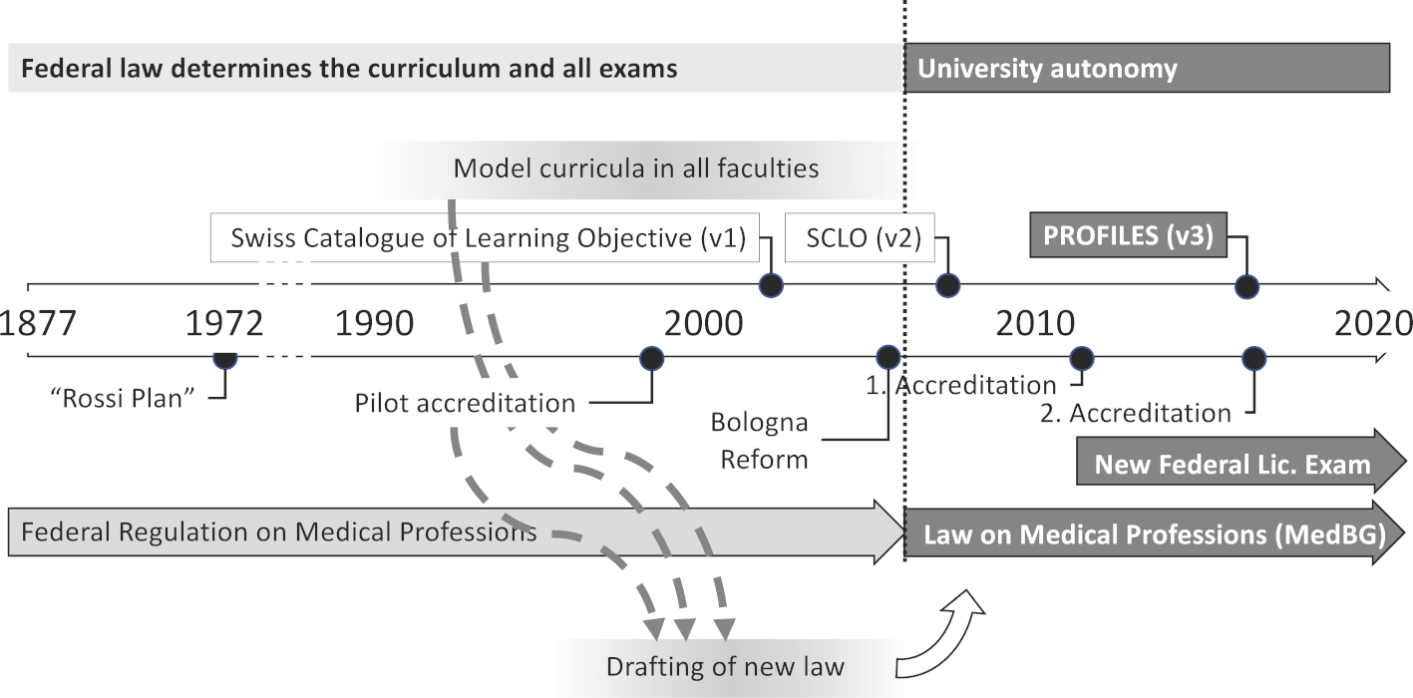
Evolution of the framework conditions of Swiss medical education.

Towards the end of the 1980s, dissatisfaction with the offered undergraduate medical training grew among students, lecturers and postgraduate education centres. Discussions at the SVMA (Swiss Association of Medical Education) and at the Swiss Medical Interfaculty Commission (*SMIFK, an advisory body composed of representatives of all Swiss faculties and the federal authorities involved in the undergraduate and postgraduate medical education*) increasingly led the faculties to rethink their curricula and to restructure them to different extents, including new types of exams. This meant that the Federal Office of Public Health (FOPH) had to approve so-called derogations to the existing legislation – similar to the “Model Study Course Clause” included in the Medical Licensure Act in Germany in 1999 – to permit changes to the curriculum. This allowed the medical faculties to introduce more self-determined curricula, mostly with organ- and system-based interdisciplinary modules, as well as problem-oriented learning (wholly or in hybrid form with accompanying lectures), with local variations at the various faculties. By 2002, all faculties had submitted derogations and implemented their local undergraduate training model. However, this experimental phase was not sustainable, and the paths towards reform that had been taken were too divergent to be regulated uniformly under existing law. As a result, the FOPH arranged for a complete revision of federal legislation, which devolved training sovereignty to the universities without giving up federal control of medical education. Three instruments were provided for in the new law, which were developed and tested by SMIFK over time: an accreditation procedure for medical degree courses, a Swiss catalogue of learning objectives and a revised federal examination format to regulate admission to postgraduate education. The reform efforts of the faculties in the 1990s ultimately led to far-reaching legal reform and to the implementation of the three instruments mentioned above, which paved the way for the wide-ranging reforms of medical studies in Switzerland in the 2000s (see figure 1 (Fig. 1)).

#### 3.2. Description of the different federal projects and legislative elements

##### 3.2.1. The new Law on Medical Professions (MedBG)

Two of the proposed instruments, namely the introduction of a new Federal Exam and the accreditation of undergraduate curricula – especially concerning quality assurance – were included in the so-called Law on Medical Professions (MedBG). This Federal Law concerning university medical professions (human medicine, dentistry, chiropractic, pharmacy and veterinary medicine) came into force in September 2007. It regulates university undergraduate and postgraduate education, and the continuous medical training and practising of university medical professions throughout Switzerland. Concerning human medicine degrees, the MedBG defines the requirements for undergraduate education, as well as the prerequisites for graduating (the Federal Diploma). The law thus provides a framework within which the medical faculties have much leeway in designing their curriculum.

The catalogue of learning objectives developed by the SMIFK was adopted by the federal regulation of the federal exam of university medical professions, to define the content of the federal exam.

The law no longer determines the exam (and curriculum structure) of undergraduate medical education, but establishes three tools to control the quality of training. These three instruments and their implications for medical degree courses are presented below.

##### 3.2.2. The introduction of programme accreditation

The intense discussions about the quality of education that took place in the wake of the study reforms of the 1990s raised the issue of accreditation. The decisive factor for its practical implementation was a request from the US Liaison Committee on Medical Education (LCME) for an established accreditation procedure in Switzerland. 

In 1999, a non-compulsory pilot accreditation of the training programmes was carried out at all Swiss medical faculties based on an internal (self-evaluation) and external evaluation (from an international group of experts) process. The pilot accreditation was prepared and accompanied by a joint SMIFK working group. This approach led to two main outcomes. On the one hand, it stimulated the faculties to make profound adjustments, such as a greater emphasis on General Practice, clearer curriculum management and the explicit development of educational goals. On the other hand, the procedure was perceived as an important quality assurance instrument and was therefore included in the MedBG, such that only students from an accredited training programme are admitted to the Federal Exam. The programmes now have to be accredited every seven years. The accreditations were conducted by the Swiss Agency for Accreditation and Quality Assurance (AAQ) at all locations initially in 2011 and in a second accreditation cycle in 2018. 

##### 3.2.3. The Federal Exam

Art. 14.2 of the MedBG stipulates that the Federal Exam must establish whether students:

1. possess the specialised knowledge, skills and abilities as well as the behaviour and social skills required to practise the corresponding medical profession; and
2. meet the requirements for the required postgraduate training.

This new Federal Exam is far more than a final university exam (a function achieved with the master’s degree and is considered a prerequisite for taking the Swiss Federal Exam) and functions as a professional entrance exam for postgraduate medical training [1]. Based on extensive collaborative preparatory work between the FOPH, the faculties and the Institute of Medical Education of the University of Bern, the two-part federal licensing exam was implemented in its new form in the summer of 2011 [2], [3]. The written part is conducted through multiple-choice (MC) questions, divided into two exams with 150 questions in each, using an interdisciplinary, case-related and practice-oriented approach. The practical clinical exam – Clinical Skills (CS) – is an OSCE-based exam in which students have to pass through 12 stations, for 15 minutes each, including rotation time. Standardised patients at all stations and assessment is carried out by trained examiners. Both exam parts are carried out simultaneously at all Swiss educational sites and are supported and evaluated centrally by the Institute for Medical Education (IML). For the OSCE exam, a multi-level quality assurance programme is carried out. Furthermore, quality assurance and further development projects are regularly performed, to sustainably guarantee high exam quality.

##### 3.2.4. The catalogue of learning objectives 

The catalogue of learning objectives was compiled by the SMIFK in cooperation with more than 100 lecturers from all medical faculties and, after consultation with medical societies, was introduced in 2002 and included in the new Federal Exam regulation [4]. Four main reasons led to the creation of this catalogue.

1. The regulation that previously controlled medical training only prescribed a few general objectives and exam topics; as a result, the curriculum reforms in the 1990s were carried out without explicit and shared learning objectives.
2. The 1999 pilot accreditation identified the lack of training objectives as a weakness of Swiss undergraduate medical training.
3. The then imminent bilateral agreements between the European Union and Switzerland also aimed to establish mutual recognition of university qualifications. In anticipation of the ratification of these treaties, it became essential for the medical faculties in Switzerland to set common goals that were compatible with the directives of the European Union.
4. A preliminary draft of the new law on medical education was published in 1999. It defined general objectives of medical education and allowed the development of the learning objectives that would be compatible with future law.

This Swiss learning objective catalogue was partially revised in 2008 (introduction of CanMEDS roles, reduction in the number of discipline-specific learning objectives) and entirely revised in 2017 (alignment with CanMEDS 2015, introduction of nine Entrustable Professional Activities (EPAs), abolition of all discipline-specific learning objectives) [5]. The resulting 34-page document called PROFILES (Principal Relevant Objectives and Framework for Integrative Learning and Education in Switzerland, http://www.profilesmed.ch/), represents the frame of reference for undergraduate medical education. It focuses on roles (competencies), tasks and situations that might be required of a resident on their first day of postgraduate training and which he/she is expected to handle without direct supervision [6]. Especially since the second edition, this document has played a central role in guiding the training content of faculties. The strong re-orientation of the third edition (PROFILES), has led to profound adjustments being made within all faculties.

##### 3.2.5. The Bologna Reform in medical degree courses

In addition to the three elements that were specific to medical education, the implementation of the Bologna system was another important structural element for medical degree courses in Switzerland. Following a decision by the Rectors’ Conference, the Bologna system was introduced between 2006 and 2007 for all university and technical college programmes, including medical studies. This splits medical studies into a bachelor’s and a master’s degree each lasting three years. Successful completion of a master’s degree is a prerequisite for registration for the Swiss Federal Exam [7]. Even though the Bachelor Degree in Medicine in Switzerland has not gained any importance as a qualifying degree for vocational or further education, this division has brought bachelor’s–master’s flexibility into medical education. This is reflected in various measures to increase the number of study places in Switzerland, including the current special programme in human medicine. At the same time, the bachelor–master interface is becoming increasingly important as a starting point for the establishment of partial bachelor’s or master’s programmes and as an interface for student mobility at this point between existing and new partial degree programmes. 

A written scientific master's thesis also had to be introduced into the master's programme as a requirement for all students. The master's degree is followed by an optional doctoral thesis. The introduction of a Master of Medicine degree probably also favoured the distinction between the responsibility for university education (with its own university degree) and the FOPH's responsibility to guarantee the qualification of physicians who embark on clinical postgraduate training (with a federal licensing exam).

#### 3.3. Specific development of individual curricula

Four undergraduate programmes with their location-specific characteristics are presented below, as examples of the development of curricula and their model approaches against the background of the dynamic framework offered by the various federal Swiss projects and laws. 

##### 3.3.1. University of Fribourg (Switzerland)

Until 2008, the Faculty of Science and Mathematics of the University of Fribourg offered the first two years of medical studies as a bilingual curriculum (French and German). The students were then able to complete their medical studies in one of the five other medical faculties. With the introduction of the Bologna Reform in 2007, a third year was introduced, to be able to offer a complete bachelor’s degree. A hybrid curriculum based on that of the other faculties, with a high proportion of clinical and practical training, ideally prepares the students for entering the master’s programmes of the other Swiss faculties. In recent years, the Canton of Fribourg has joined efforts to address the Swiss shortage of doctors and has decided to offer a master’s programme so that students can complete their entire medical degree in Fribourg. Due to PROFILES and the new federal examination, Fribourg will be able to offer 40 students a master’s programme with a focus on family medicine and social accountability from 2019. With an uncompromising programmatic exam design [8], based on a progress test, an e-portfolio and learning advisors, the master’s programme fully exploits the existing freedom of education design. To implement this new master’s programme, a chair for Medical Education was created, which unites and expands the existing pedagogic resources.

##### 3.3.2. University of Geneva

In 1995, as part of a comprehensive reform of the curriculum, a new curriculum was implemented in Geneva, which deviated from a traditional curriculum and moved towards an integrated overall concept, composed of interdisciplinary modules on the organ systems of the human body, using problem-based learning in small groups from the second academic year onwards [9], [10]. The first year focused on basic medical science, and some purely biological subjects (such as plant biology) were abolished and a programme related to humans, health and the community was introduced.

A longitudinal programme on clinical competencies was also integrated into the modules, including doctor–patient communication training [11]. Another longitudinal programme on collaborative and ethical dimensions allows students to acquire skills in these areas. A so-called "learning unit for immersion in health care at the community level" enables students to carry out a community project outside of the university, in Geneva or at another location [12]. Furthermore, a programme for medical humanities allows students to develop an interest in additional areas of health care.

Hands-on experience in out-patient medicine is introduced in the second year through regular, all-day visits to GP and paediatric surgeries.

From the second year onwards, an additional programme of optional activities allows students to deepen their knowledge of clinical or non-clinical disciplines.

Clinical training begins in the fourth year of studies, with the immersion of students in clinical rotations, accompanied by practical and theoretical teaching. Theoretical knowledge is mainly covered in small case-based learning groups, in which clinical reasoning is reproduced and acquisition of the corresponding knowledge is promoted. The sixth and final year contains a full clinical programme in the form of an elective year. Students choose disciplines from a catalogue covering the French part of Switzerland, although doing internships abroad is, of course, also possible.

To facilitate the entry of students into the clinical environment, an introductory learning unit for clinical approaches has been introduced. The aim is to optimise the transition from the pre-clinical years in which students learn the mechanisms, to the clinical years in which they are trained to solve clinical problems [13].

In the Swiss federal context, the Faculty of Medicine of the University of Geneva is also a pioneer in the use of simulation as well as simulated and standardised patients in the training and assessment of its students.

The changes made over the years mainly concern the adaptation of the syllabus to the requirements of the Bologna system and to those of the Federal Exam, which conveys more autonomy in student assessment to the faculties. In this context, the master’s thesis has been introduced between the fourth and fifth academic years to enhance the status of scientific work and critical analysis. In addition, an interprofessional simulation centre was set up in 2013 to provide continuous development of a longitudinal interprofessional curriculum for medical students and students from the health professions of the partner University of Applied Sciences Health [14].

For this reform, a medical education unit (Unit of Development and Research in Medical Education, UDREM, https://www.unige.ch/medecine/udrem/en/) was founded in 1994, the missions of which are the conceptual support to the medical curriculum, the education and training of teachers, the evaluation of teaching and learning activities, and research in the field of medical education. Over the years, this unit has played an important role in supporting innovation and implementing new concepts.

##### 3.3.3. University of Zurich

In addition to a degree in dentistry and a specialised master’s degree course in chiropractic medicine, the Faculty of Medicine of the University of Zurich (UZH) offers a six-year undergraduate programme in human medicine according to the Bologna model. The main goal of the essentially competency-based curriculum is to train excellent physicians for the Swiss health care system. Due to the further increase in the number of students in the 2017 semester, 300 students are currently studying in years two to six, and in the first year of study, there are already 372 students of human medicine (including up to 20 students of chiropractic medicine). The programme is divided into a bachelor's and a master's degree course of three years each and combines in-depth horizontal integration (interdisciplinary study areas and topics) with a moderate and increasing vertical integration of clinical content during studies. 

In the context of the reform of medical degree courses in Switzerland, Zurich offers one unique feature regarding the establishment of a partial model study course: in addition to core studies, the UZH Medical Faculty curriculum offers compulsory elective subject areas ("elective degree courses") during the first four years of study, in which students can currently select one module per semester out of about 50 modules on offer. The main objectives of the elective degree courses include:

1. Deepening core studies content.
2. Teaching selected medically relevant content that extends beyond core studies and the specifications of the learning objective catalogue.
3. Creating opportunities to set personal priorities in medical studies.

The subject areas of the elective degree courses (in the second to fourth year of study in human medicine and dentistry) cover almost the whole breadth of medicine: clinical medicine, basic biomedical sciences, population research, humanities and the translational linkage of these sciences. The workload of each module carries 4 ECTS credit points. This constellation enables the targeted and early development of students in specific (subject) areas, without giving up the generic qualification (qualification for general continuing education). In addition to the free-choice modules, so-called focus programmes are particularly important, which give students the opportunity to focus on areas of personal interest during their studies. The elective degree courses can currently count towards a structured study focus programme (“track”) in three areas:

- “Psychiatry and Psychotherapy”
- “Medicine and Technology (in collaboration with the ETH Zurich)
- “Research in Medicine”

For these tracks, the master’s thesis, a mentoring programme and three months of the elective year must be completed in addition to the elective degree courses. This means that 60 ECTS credits can ultimately be earned in the area of focus, which will be recognised in the form of a certificate. For the psychiatry track, the possibility of credits counting towards subsequent postgraduate specialisation is currently being discussed. This curricular model offers great potential to strengthen the continuum of undergraduate and postgraduate education [15]. 

The Education Network for Medicine, founded in 2017, should also be mentioned in this context, which offers excellent potential in this framework to further differentiate competency profiles as well as to develop new teaching methods and teaching and learning settings [https://www.medunet.ch/]. 

##### 3.3.4. University of Bern

The Faculty of Medicine of the University of Bern also offers a six-year university degree in medicine. The bedside teaching of small groups introduced in 1973 was renamed Clinical Skills Training (CST) in the 1990s, and a guideline for students and lecturers with learning objectives was written for each of the 13 teaching modules. 

This ensured compulsory teaching, which since 2003 has been tested in an interdisciplinary summative OSCE in the third year of study. One specific feature introduced in 1973 in Bern was clinical rotation blocks in the fourth and fifth years of study. Over 28 weeks, all students completed hospital internships in five disciplines (eight weeks in internal medicine, six weeks each in surgery and paediatrics and four weeks each in psychiatry and gynaecology/obstetrics) and additionally took three-week-long courses each in ophthalmology, dermatology and ENT. No lectures were held during these hospital internships, which extended over 13 months at a time. In addition to these clinical rotation blocks, the elective year (analogous to the German practical year) was also required. The medical degree course in Bern was considered to be the most practice-oriented degree course in Switzerland at the time.

In 1999, another curriculum reform took place; in addition to the number of university places being halved from around 250 to 125, the first three years of study were transformed into a problem-oriented problem-based learning (PBL) hybrid curriculum. In the new PBL curriculum, lecture time was reduced to about 8-10 hours per week and two PBL tutorials per week were introduced, with groups of 8-10 students. Since then, the maximum number of classroom teaching hours per week has been 20. This leaves the students plenty of time for self-study.

Within the scope of the above-mentioned current projects and legislative elements, the undergraduate medical programme in Bern was split in 2007 into a more strictly regimented bachelor's and a master's programme and ECTS credits were introduced. A longitudinal internship with GPs was also introduced throughout the medical degree course [16]. Over 600 general practitioners supervise students from the first to the fifth academic year directly in their practices in 1:1 tutoring. For the introduction of a master’s thesis, the Bern clinical rotation blocks were reduced from 28 to 20 weeks. At the same time, a didactic concept with student assessments was specified for the participating teaching hospitals. Since 2011, students have been required to carry out formative, workplace-based assessments (mini-CEX, DOPS and presentations) in their elective blocks. Teaching contracts are signed with external hospitals, which are also financially compensated for teaching students.

Since 2009, a total of six different communication courses have been introduced and further developed in the Bern Interdisciplinary Skills and Simulated Patient Center (BiSS), which was expressly set up for this purpose in 2011 [17]. Courses include feedback training in role-play in the first year of studies, general communication training in the fourth year of studies and specific communication training in the sixth year of studies – each with video recordings of all conversations. Further courses are training for telephone communication, anaesthesia communication training and geriatric communication/assessment training, each using simulated patients. Formative OSCEs were also introduced in the third and sixth year of studies [17]. 

Forward-thinking interprofessional courses, such as the peer-taught venipuncture course that has now become mandatory at all three institutions involved (the University of Bern, Bern University of Applied Sciences and the Centre for higher Education in Nursing) and an interprofessional case-based seminar on confidentiality was developed, which has since been integrated into compulsory education [18]. Since 2018, a peer-taught sonography course has been offered to all students. 

Since 1999, the number of places has increased gradually, with a cohort size of 320 first-year students in the autumn semester of 2018, although further increases will require further reforms and more space. This includes a new building for teaching (2,500 m^2^) with a new BiSS, an anaesthesia simulation centre and a learning centre, which have just opened in a refurbished former city hospital. In Bern, the Institute for Medical Education (IML) has supported teaching development, examinations and further innovative developments in Bern’s medical degree courses since 1972. In addition, the IML performs federal tasks, such as supporting the Federal Medical Exams and conducting research in medical education.

## 4. Discussion

The current landscape of undergraduate medical education in Switzerland is the result of profound changes that have taken place over the last 20 years (see figure 1 (Fig. 1)). These began in the 1990s with the increasing dissatisfaction among students, teachers and universities regarding the offered undergraduate training, which was controlled through prescribed exams at the federal level. Thanks to derogations, a period of experimentation ensued, in which each university planned and implemented its approach and vision of medical education. This period of experimentation illustrated how different the needs of each university were. To meet these needs, the FOPH has wisely decided to rewrite the 1877 Federal Act on undergraduate Medical Education, based on the insights gained. 

However, a way had to be found for the FOPH to fulfil its role as a guarantor of the quality of the training of future doctors who have access to the Swiss medical labour market, while simultaneously delegating responsibility for providing this basic training to the universities. In a collaborative approach, the various stakeholders (FOPH, SMIFK and the medical faculties) proposed three instruments to achieve this goal: an accreditation of the undergraduate programmes, a federal licensing exam (which regulates admission to clinical specialisation) and with PROFILES, a frame of reference to define the competencies and skills that are expected on the first day of specialisation. 

This new law came into force in 2007 and was able to achieve the desired flexibility without sacrificing quality [2], [19]. The universities used the autonomy gained, to redesign their courses with great freedom and to set priorities or to introduce innovative types of exams; in particular, more formative and practical clinical exams were introduced. New challenges, such as an increase in the numbers of study places, could also be tackled more flexibly as a result. Quality continued to be guaranteed by the FOPH. 

The accreditation process allows the programmes to be screened and self-reflection on behalf of the faculties as a result of the self-assessment report that has to be completed is particularly valuable. Unfortunately, the same standards were not used in the last two accreditations; moreover, the composition of the expert review groups for external evaluation is different for each faculty. 

The Federal Exam allows the outcomes of undergraduate training to be compared across Switzerland and to secure the competencies of the residents admitted to postgraduate training. Interestingly, no significant differences were found in exam performance between the widely different curricula [2]. Although the pass–fail thresholds are set relatively low (resulting in 0–3% failures per faculty and year), these examinations have a very positive influence on the learning behaviour of the students. For the first time, the entire study content, at least of the clinical phase, is learnt integrally.

The implementation of the new learning objectives, according to PROFILES, represents a significant change for each faculty. Learning objectives that had previously been well integrated into the curriculum mapping (clinical pictures) have disappeared, with EPAs having to be implemented instead. PROFILES and especially the EPAs represent merely a framework, not a detailed catalogue of items. As a result, it requires major effort for each faculty to derive concrete educational objectives; however, much freedom remains.

An important factor in improving and innovating teaching is the financial compensation of teaching in a clinical setting, which is an aspect not explored in this study. The competition in clinical and research activities and the maximisation of services for patient care are pushing teaching activities to the margins. This represents a considerable challenge for all Swiss medical faculties.

## 5. Conclusion

Politicians clearly assigned responsibility for medical training to the Federal Office of Public Health and not to the State Secretariat for Education, Research and Innovation, which is otherwise responsible for university education. 

This facilitated stringent legislation at the national level and allowed for coherence between education, specialisation and continuing education. Within this framework, the faculties have the freedom and responsibility to train students. A critical beneficial factor is the open, institutionalised and collaborative exchange between the faculties, the responsible Federal Office of Public Health and the other stakeholders.

The federal licensing exam, based on the PROFILES catalogue of learning objectives, creates a binding framework for the training of physicians. In the design of the specific learning objectives, the faculties possess much room for manoeuvre, but a degree of uncertainty exists regarding the exam content of the licensing exam. The intensive cooperation between the Federal Government and the faculties has created a relationship of trust that outweighs this insecurity. Moreover, much experience has been gained over the last ten years. Despite changes to the learning framework – it is no longer a learning objective catalogue – the content of the licensing exam will not change profoundly. 

The impending challenge today of introducing the EPAs prescribed by PROFILES across Switzerland draws on this close and dynamic cooperation between the faculties and the FOPH. The new framework created over the last 20 years ensures an efficient and timely transformation of medical education.

## Competing interests

The authors declare that they have no competing interests.
